# The perioperative transition of serum biomarkers of a 1.5-year-old boy with very-long-chain acyl-CoA dehydrogenase deficiency

**DOI:** 10.1016/j.ymgmr.2021.100760

**Published:** 2021-04-19

**Authors:** Ryosuke Bo, Hiroyuki Awano, Kenji Yamada, Mayu Ooi, Yuichi Okata, Yuko Bitoh, Satoshi Mizobuchi, Kazumoto Iijima

**Affiliations:** a Department of Pediatrics, Kobe University Graduate School of Medicine, 7-5-1 Kusunoki-cho, Chuo-ku, Kobe 650-0017, Japan; b Department of Pediatrics, Shimane University Faculty of Medicine, 89-1 En-ya-cho, Izumo, Shimane 693-8501, Japan; c Department of Anesthesia, Kobe University Graduate School of Medicine, 7-5-1 Kusunoki-cho, Chuo-ku, Kobe 650-0017, Japan; d Department of Pediatric Surgery, Kobe University Graduate School of Medicine, 7-5-1 Kusunoki-cho, Chuo-ku, Kobe 650-0017, Japan

**Keywords:** VLCAD deficiency, Perioperative management, Fatty acid oxidation, Newborn screening, Volatile anesthetics, Tetradecenoylcarnitine, 3-OHB, 3-hydroxybutyrate, CK, creatine kinase, FFA, free fatty acid, NBS, newborn screening, VLCADD, very long-chain acyl-coenzyme A dehydrogenase deficiency

## Abstract

Very long-chain acyl-coenzyme A dehydrogenase deficiency (VLCADD, OMIM 201475) is a congenital fatty acid oxidation disorder. Individuals with VLCADD should avoid catabolic states, including strenuous exercise and long-term fasting; however, such conditions are required when undergoing surgery. The perioperative management of VLCADD in infants has rarely been reported and details regarding the transition of serum biomarkers reflecting catabolic status have not been disclosed. Herein, we present the perioperative clinical and biological data of cryptorchidism in a 1.5-year-old boy with VLCADD. The patient was diagnosed through newborn screening and his clinical course was very stable. Genetic testing of *ACADVL* revealed compound heterozygous variants c.506 T > C (p.Met169Thr) and c.606-609delC (p.L216*). The enzyme activity of the patient with VLCAD was only 20% compared to that of healthy control. Left orchiopexy for the pediatric cryptorchidism was planned and performed at 1 and a half year of age. Induction anesthesia involved thiopental, fentanyl and rocuronium. The glucose infusion rate was maintained above 6.6 mg/kg/min starting the day before surgery until the operation was completed. Anesthesia was maintained with sevoflurane at approximately 2%. The serum concentration of tetradecenoylcarnitine were stable during the operation, ranging between 0.08 and 0.19 μM (cutoff <0.2 μM), and never deviated from the reference range. Concentration of other serum biomarkers including free fatty acid, 3-OH-butyrate, and creatine kinase, remained similarly unchanged. In this report, we describe the uneventful perioperative management of unilateral orchiopexy for left cryptorchidism in a 1.5-year-old boy with VLCADD using sufficient glucose infusion and volatile anesthesia.

## Highlights

• Catabolic biomarker status details during surgery in infants with VLCADD is lacking.
• We performed uneventful surgical intervention in a 1.5-year-old patient with VLCADD.
• Serum levels of tetradecenoyl carnitine remained stable during the operation.
• Serum biomarkers creatine kinase, 3-hydroxybutyrate, and free fatty acid remained similarly unchanged.
• These findings will be beneficial in the management of VLCADD.

## 1 Introduction

Very long-chain acyl-CoA dehydrogenase deficiency (VLCADD, OMIM 201475) is a congenital fatty acid oxidation disorder with a prevalence of approximately 1:93,000 births in Japan [1]. Clinical symptoms for patients with VLCADD are provoked during catabolic conditions, such as infection, exercise, and fasting, and can include severe hypoketotic hypoglycemia, liver dysfunction, cardiac involvement, rhabdomyolysis due to the accumulation of long-chain acyl-CoA and acylcarnitines, and a shortage of energy [2,3]. Avoiding fasting and the consumption of a diet restricted in long-chain fatty acids, a diet rich in middle chain triglycerides (MCT), like MCT milk, or oil are recommended as chronic therapy to prevent catabolic situations [4,5].

One concern for patients with VLCADD is the need for surgery for other medical conditions. Surgical procedures require perioperative management, including prolonged fasting, physical burden, and certain anesthetics that may include the use of lipid emulsions, which can lead to metabolic derangements [6]. Although there have been reports that focus on perioperative management of patients with VLCADD in adulthood [7,8], anesthesia of infant patients with VLCADD has rarely been reported [6,9]. Furthermore, the perioperative use of some medications is controversial. For instance, propofol, which includes lipid emulsions, has been reported to interfere with mitochondrial function [10] and volatile anesthesia may cause elevated free fatty acid (FFA) concentrations [6,8].

Long-chain acylcarnitines could be a reliable biomarker for metabolic status before any increase is detected in the concentrations of other biomarkers; however, immediate acylcarnitine analysis is not always available [11]. Therefore, it is recommended for many facilities that serum creatine kinase (CK) concentrations correlated with long-chain acylcarnitines should be used as biomarkers during the acute phase of fatty acid oxidation disorders [12,13]. However, changes in concentrations of these serum biomarkers during surgery has not been determined. As a result, inferring the extent of the burden that perioperative procedures may have on a patient remains impossible.

In the current study, we performed a surgical course to treat cryptorchidism in a 1.5-year-old boy with VLCADD. Detailed perioperative clinical and biological findings regarding long-chain acylcarnitines and other serum biomarkers were clarified. The findings were helpful in the perioperative management of VLCADD.

## 2 Material and methods

### 2.1 Case details

A 1-year 6-month-old Japanese boy diagnosed with VLCADD was enrolled in the current study. He was the second child of non-consanguineous parents born at 39-weeks 5-days of gestation, weighing 3440 g, and had a healthy elder sister. Elevated concentrations of tetradecenoyl carnitine (C14:1) (0.46 μM; cutoff, <0.27 μM) and C14:1/acetylcarnitine (C2) ratio (0.019; cutoff, <0.013) were detected during newborn screening (NBS). Serum acylcarnitine analysis also suggested the elevation of C14:1 at 0.35 μM (cutoff <0.2 μM) 9 days after birth. Genetic testing of *ACADVL*, the gene encoding VLCAD, revealed compound heterozygous c.506 T > C (p.Met169Thr) and c.606-609delC (p.L216*) variants, the former of which was a novel variant. The enzyme activity of VLCAD was only 20% compared to that of healthy control. Ultimately, the patient was diagnosed with VLCADD at the preclinical stage. After the diagnosis, no obvious clinical symptoms or laboratory abnormalities were shown except for the elevation of C14:1. MCT-supplemented medical formula was administered only during the acute phase of upper respiratory infections, but l-carnitine treatment was not started as his free carnitine concentrations were within the normal range.

Cryptorchidism of the left testis was identified at the one-month medical check-up. Left orchiopexy for the cryptorchidism was planned and then performed at 1 year 6 months of age. Informed consent was obtained from the parents of the patient.

### 2.2 Monitoring of laboratory data

We collected a total of five blood samples, including a preoperative sample, a post-induction sample, and samples 30 min, 60 min, and one day after surgery. Blood samples were taken using an arterial line to reduce the effects of hemolysis. The results of plasma glucose and biological tests, including serum concentrations of CK, 3-hydroxybutyrate (3-OHB), and FFA were also measured at the same timing. The plasma glucose, CK, 3-OHB, and FFA were measured using ADAMS Glucose GA-1171 (Arkray,Inc. Kyoto, Japan), Cygnus Auto CK (Shino-Test Corporation, Tokyo, Japan), 3HB-L (Kainos Laboratories, Tokyo, Japan), and NEFA-HR (FUJIFILM Wako Pure Chemical Corporation, Osaka, Japan), respectively. Serum acylcarnitine concentrations during surgery were analyzed via tandem mass spectrometry (MS/MS) using non-derivatized acylcarnitine analysis kit (NeoSMAAT®AC: Sekisui Medical Corporation, Tokyo, Japan).

### 2.3 Enzyme assay

The residual VLCAD enzyme activity was measured using fibroblasts or lymphocytes, as reported previously [14].

## 3 Results

The milk and food intake was stopped 6 h before surgery. Glucose infusion rates were maintained above 6.6 mg/kg/min from the day before surgery until the operation was completed. No abnormalities were detected on echocardiography or electrocardiography before general anesthesia.

**Fig. 1:**
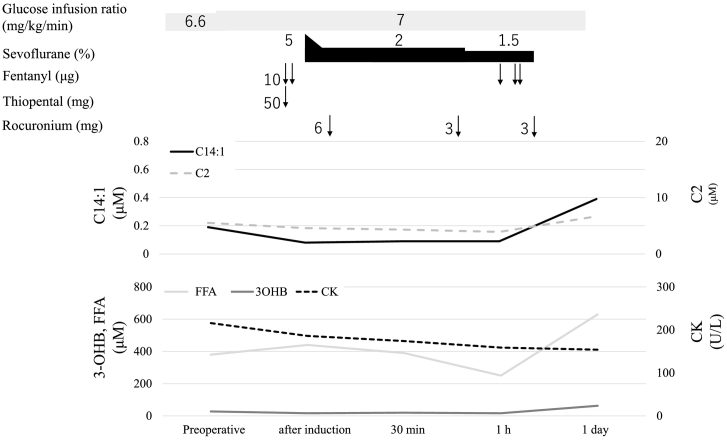
Perioperative transition of serum biomarkers, including acylcarnitine concentrations. C14:1, tetradecenoylcarnitine; C2, acetylcarnitine; 3-OHB, 3-hydroxybutyrate; FFA, free fatty acid; CK, creatine kinase.

Induction of anesthesia at the start of the operation included thiopental (5 mg/kg), fentanyl and rocuronium as shown in Fig. 1. Anesthesia was maintained with approximately 2% sevoflurane. Careful monitoring of electrocardiography and frequent monitoring of glucose and serum biomarkers, such as CK, 3-OHB, FFA, and serum acylcarnitines, were performed before and every 30 min after the surgery. Hydration including glucose was continued post-surgery until normal oral intake was observed. Plasma glucose levels were stable, ranging between 5.3 and 7.1 mM (reference value, 4.0–6.0 mM). As shown in Fig. 1, CK concentrations were 159–216 U/L (reference value 59–248 U/L), FFA concentrations were 250–380 μM (reference value 100–810 μM), and 3-OHB concentrations were 17–28 μM (reference value 28–120 μM), all of which were within normal ranges. Similarly, serum C14:1 concentration was stable during the operation, ranging between 0.08 and 0.19 μM (cutoff <0.2 μM) and never deviating from the reference range. Induction and maintenance of anesthesia did not elevate C14:1 concentration. The total length of surgery was 67 min and the duration of anesthesia was 152 min. The preoperative and postoperative course was uneventful with no abnormal biological findings or clinical symptoms during glucose infusion and strict monitoring. The day after the operation, C14:1 concentration was relatively high (0.39 μM), but the patient was able to leave the hospital without any problems.

## 4 Discussion

In the current study, we describe the successful perioperative management of unilateral orchiopexy for left cryptorchidism in a 1.5-year-old boy with VLCADD using sufficient glucose infusion and volatile anesthesia. This is the first report showing the detailed transition of serum biomarkers under general anesthesia, including acylcarnitines, CK, 3-OHB, and FFA.

During the operation, we performed strict monitoring of the biological markers of VLCADD at the preclinical stage. In the past, when NBS for VLCADD did not exist, postoperative death of a patient with undiagnosed VLCADD was reported [15]. Subsequently, VLCADD has been commonly diagnosed during the pre-symptomatic stage through NBS, making it possible to closely monitor these patients during surgical procedures. However, it remains difficult for attending physicians to determine whether surgical stress causes a metabolic decompensation in patients with pre-symptomatic VLCADD. Due to the shortage of evidence on the perioperative management of VLCADD, extreme caution is required when performing surgery, even if it is over-managed.

In this study, we determined the concentrations of C14:1 as a reflection of catabolic status and found that they remained unchanged during surgery under sufficient glucose infusion. Similarly, concentrations of other serum biomarkers, including CK, FFA, and 3-OHB, were within normal ranges and remained stable. This suggested that sufficient glucose infusion and close monitoring could prevent metabolic derangement, even in infants with VLCADD. Elevation of C14:1 and CK concentrations were observed the day following the surgery, probably because we stopped hydration with glucose after assuring a normal meal intake. Previous studies have shown that catabolic situations are not limited to the day of the surgery [6,16]. Accordingly, it is important for attending physicians to focus on catabolic status even after surgery.

Cryptorchidism is a relatively common condition in infants; therefore, it can occur as a complication in individuals with VLCAD deficiency. From the perspective of pediatric surgeons, surgical intervention for cryptorchidism is recommended at 6 months, and no later than 18 months, as delayed surgery may cause an irreversible pathological change [17,18]. As the current patient had VLCADD, the timing of the surgical intervention was delayed as much as possible. The stable clinical condition of the patient and concentration of the serum biomarkers during the procedure suggested that the surgery did not result in metabolic decompensation in this young patient. However, to confirm this finding as an expected outcome, more cases are needed.

In the current case, we selected sevoflurane for maintenance of anesthesia, and could ultimately perform an uneventful surgical intervention to treat cryptorchidism. Propofol includes lipid emulsions; and therefore, it can impair mitochondrial entry of long-chain fatty acids and inhibit the respiratory chain at several points [6,8]. However, there are case reports demonstrating the uneventful use of propofol in individuals with VLCADD and other fatty acid oxidation disorders [7,19]. This makes the use of propofol in patients with VLCADD controversial. Similarly, it has been reported that volatile anesthetics are associated with significant increases in FFA concentrations during the first phases of anesthesia, which in the past has resulted in the development of rhabdomyolysis [6]. Our results are consistent with the results of recent publications that reported relatively safe volatile agents [7,8].

The current study had a limitation. Our patient harbored a novel *ACADVL* mutation and his residual VLCAD enzyme activity was relatively high, suggesting his phenotype may have been asymptomatic or benign. Whether metabolic derangement occurs or not following anesthesia depends on several factors in addition to the *ACADVL* genotype, including the duration, the degree of surgical intervention, and surgical-related stress. Therefore, this strategy may not be well suited to manage the perioperative period for more severe cases of VLCADD or those involving more invasive surgery. More cases, including those with more diverse phenotypes or known genotypes are needed.

## 5 Conclusion

Adequate glucose infusion and careful monitoring can lead to uneventful surgical outcomes and stable biomarkers in patients with VLCADD, even in infants.

## Funding

This research was partially supported by Grant Number 19K17298 (Chief Investigator: Ryosuke Bo) from the 10.13039/501100004726Ministry of Health, Labor and Welfare of Japan. The sponsor had no role in the study design; in the collection, analysis, or interpretation of the data; in the writing of the report; or in the decision to submit the article for publication.

## Declaration of Competing Interest

The authors declare that they have no conflicts of interest.
